# FEM Numerical and Experimental Study on Dimensional Accuracy of Tubes Extruded from 6082 and 7021 Aluminium Alloys

**DOI:** 10.3390/ma16020556

**Published:** 2023-01-06

**Authors:** Dariusz Leśniak, Józef Zasadziński, Wojciech Libura, Krzysztof Żaba, Sandra Puchlerska, Jacek Madura, Maciej Balcerzak, Bartłomiej Płonka, Henryk Jurczak

**Affiliations:** 1 Faculty of Non-Ferrous Metals, AGH University of Science and Technology, 30-059 Kraków, Poland; 2 Łukasiewicz Research Network—Institute of Non-Ferrous Metals, 44-100 Gliwice, Poland; 3 Albatros Aluminium Corporation, 78-600 Wałcz, Poland

**Keywords:** AlZnMg alloys, extrusion, porthole dies, metal flow, die deflection, extrudates dimensional accuracy

## Abstract

The extrusion of hollow profiles from hard-deformable AlZnMg alloys by using porthole dies encounters great technological difficulties in practice. High extrusion force accompanies the technological process, which is caused by high deformation resistance and high friction resistance in extrusion conditions. As a result of high thermo-mechanical loads affecting the die, a significant loss of dimensional accuracy of extruded profiles can be observed. The different projects of porthole dies for the extrusion of Ø50 × 2 mm tubes from the 7021 alloy were numerically calculated and then tested in industrial conditions by using a press of 25 MN capacity equipped with a container with a diameter of 7 inches (for 7021 alloy and 6082 alloy for comparison). New extrusion die 3 with modified bridge and mandrel geometry and a special radial–convex entry to the die opening was proposed. FEM was applied to analyse the metal flow during extrusion, geometrical stability of extruded tubes and the die deflection. The photogrammetric measuring method was used to evaluate dimensional accuracy of tubes extruded in different conditions and geometrical deviations in porthole dies elements, especially the bridges and the mandrels. Research revealed a high dimensional accuracy of tubes extruded from the 6082 alloy and from the 7021 alloy by using original extrusion die 3, while much higher dimensional deviations were noted for tubes extruded from the 7021 alloy by using extrusion dies 1 and 2, particularly in relation to the circularity, centricity and wall thickness.

## 1. Introduction

The hollow extruded profiles from 7000 series aluminium alloys are currently used in a wide application of the construction of various means of transportation, including important automotive systems. Therefore, the profiles must fulfil high quality expectations, in particular, concerning dimensional accuracy. The extrusion of aluminium alloys becomes more difficult as its strength is increased. The 7000 series alloys not containing copper are easier to extrude compared to, e.g., high strength 7075 alloys, but the extrusion force is high, which is what implicates various dimensional defects in the extruded profiles. Additional difficulties are connected with a high friction at the tool surface, which produces non uniform metal flow from the die cavity. The extrusion parameters and the porthole die design can also have an impact on the extrudate geometry. The complex set of reasons of the geometrical defects in extrudate comprises the result from the unsuitable design of the porthole die and parameters of the whole production line, including extrusion process, heat treatment on the run-out table and stretching. In turn, the production parameters cover types of alloy, extrusion force, billet temperature, extrusion speed, rate of cooling in the quenching tunnel and force of stretching.

The most important tolerance measures are as follows: wall thickness, circularity and eccentricity, convexity and concavity of the walls, angularity, straightness, bow and twist along the profile. The direct reasons for the loss of profile dimensional accuracy are as follows: elastic deflection of die, including deflection of mandrels; non-uniform metal flow within the die cavity; imperfect design of the welding chamber and bearings; die wear; non-uniform temperature distribution within the billet; too high a cooling rate within the quenching tunnel; and sometimes excessive plastic deformation during stretching. When designing the extrusion process, one should take account of the fact that tighter tolerances will push up the cost and make the production longer.

When a new profile is produced, trials and corrections of the die are necessary in order to fulfil required geometrical tolerances, as well as to achieve the compromise between effectiveness, surface quality and die life. Therefore, that finite element simulation (FEM) is a useful tool for analysing the relation between the process parameters, the tool design and the profile quality.

Many researchers analysed the influence of the porthole die geometry on die deflection and the resulting geometrical quality of the extruded aluminium profiles [1,2,3,4,5]. Pinter et al. [1] analysed the influence of process parameters (ram speed, billet length and alloy) on the achieved die deformation after a determined extruded amount in the case of 6005A alloys. The authors have found that the mandrel deflection, which influences profile geometry, rises as the amount of extruded alloys increases. In the study [2], the mandrel fracture behaviour was examined through investigating the elastic deformation of the mandrel during the extrusion using FE analysis. After optimisation with the use of HyperXtrude 13.0 software, the desired die geometry giving proper material flow at the die exit and small mandrel deflection were obtained [3]. Xue et al. [4] applied different modifications of the porthole die, including two-step welding chamber to obtain uniform metal flow. An original system for the monitoring of the tool deflection, of the tool temperature and at the die bearings, as well as the pressure in the die opening, were developed and tested within [5]. The numerical simulation was applied to find the optimal die design and a proper metal flow, guaranteeing the good geometry of the product [6,7,8]. Guan et al. [9] analysed the design of a multihole extrusion die and investigated the effects of the layout of holes on the extrusion process. HyperXtrude program was used to process simulation and the die with three portholes was the optimum one, where the uniform velocity distribution, maximum welding pressure, minimum required extrusion load, and minimum die stress were obtained.

The influence of die deformation on the speed, temperature distribution and distortion of the two profiles from AA6082 alloy is reported and analysed in [10]. As a consequence of the die deflection, a bended profile with a large curvature radius was produced.

Refs. [11,12] reported that to obtain a uniform metal flow at the die exit, the porthole die was optimized by adding baffle plates on the die insert. After optimization, the concave defects on the profile were remarkably limited. Various modifications in the porthole die design were proposed by Xue et al. [4] to improve the homogeneity of metal flow from the die, giving a relative high product accuracy for a complex section from the 6060 alloy.

Hsu et al. [13] studied the varying of the welding chamber geometry and bearing length to obtain a uniform material flow. Finite element analysis and Taguchi method were used to obtain a better porthole die design for the extrusion of the 7075 alloy. Different welding chamber geometry and different length of die bearing caused metal flow to be more uniform.

Incidentally, it is worth noting that the AlZnMgCu alloys are very difficult as far as the dimensional accuracy is considered because of both the high extrusion force and deformation resistance leading to large die deflection and non-uniform metal flow. The large distortion of the rectangular hollow profile from the 6061 alloy was observed as a result of the non-uniform exit velocity [14]. To decrease this phenomenon, the second welding-chamber was applied and the bearing length and baffle block were modified in profile extrusion. In [15], the extrusion process of a large, aluminium profile for high-speed train was simulated to obtain uniform metal flow. Modifications to the porthole die, including shape of mandrels and length of the bearings, were proposed, which influenced the metal flow effectively. The baffle plates were applied in the welding chamber to balance the material flow velocity in the die cavity during the extrusion of the profile [16]. Through a series of modifications, the velocity difference in the cross-section of the profile decreased significantly. The exit velocity distribution of the profile was investigated by Chen et al. [17] by using the effects of eccentricity, the shape of the welding chamber, and uneven bearing length.

The study [18] presented 3D FEM simulations aimed at die design and process optimisation for the 7075 alloy with large differences in wall thickness. The influence of bearing length and extrusion speed on profile temperature and extrusion pressure were analysed. A longer bearing leads to a greater dimensional accuracy of the profile. In the work [19], the porthole die for the extrusion of a solid heatsink profile with high wall thickness variation was designed using finite element (FE) simulations. The structure of the die elements effectively influenced the flow behaviour of the metal. In consequence, the proposed solution can be implemented in industrial practice. The paper [20] presented the 3D FE simulation of the extrusion processes of the 7003 alloy through the porthole dies in different process variables, including billet temperature, bearing length, tube thickness and extrusion ratio. The products surface was also examined. In the work [21], the effects of the length and geometry of die land on curved profiles produced by a novel process, differential velocity sideways extrusion (DVSE), were studied through physical experiments and FEM.

In this work, the influence of different porthole die design on the metal flow during extrusion and on the dimensional accuracy of round tubes of Ø50 × 2 mm from the 6082 and 7021 alloys was investigated. The die deflection was experimentally measured for different design solutions to investigate the effect of the die deflection on the material flow. FEM simulation was applied in the design of the porthole dies and to predict the metal flow and the die deflection. The photogrammetric measuring method was used to evaluate the dimensional accuracy of tubes extruded in different conditions and the geometrical deviation of porthole dies, especially the bridges and the mandrels. The statistical correlation method was used to determine the influence of the technological parameters of the extrusion process on the dimensional accuracy of profiles. The ANOVA analysis was applied for extrusion dies.

## 2. Materials and Methods

### 2.1. Characteristics of AlMgSi and AlZnMg Alloys

**Figure 1:**
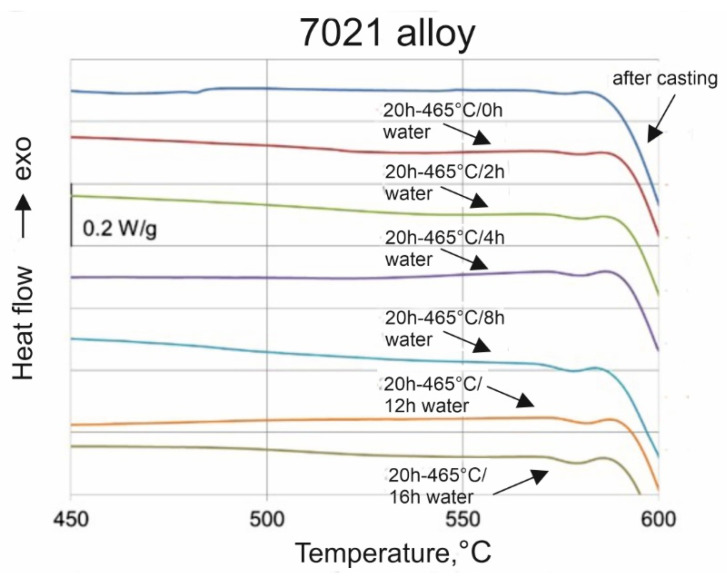
DSC curves for 7021 alloy after homogenization in different conditions.

The billets of 178 mm in diameter, of which chemical compositions are presented in Table 1 (6082 alloy) and Table 2 (7021 alloy), were DC cast in semi-industrial conditions. In the case of 7021 alloy, the low-melting microstructure components were dissolved during homogenization to a degree that was sufficient in practice—no incipient melting peaks on the DSC curves were observed (Figure 1). As a result, the significant increase in solidus temperature was obtained for 7021 alloy after homogenisation—572.1 °C with regard to the temperature of as-cast alloy at the level of 478.1 °C (Table 3).

### 2.2. FEM Numerical Modeling of Extrusion Process

The QForm-Extrusion software and specially prepared material model were used to analyse the extrusion process of tubes of Ø50 × 2 mm from aluminium alloy grade 7021 (AlZnMg) using porthole dies of various geometry. Combined Lagrangian–Eulerian approach is the method that is used in QForm-Extrusion in order to describe the deformed material motion. This approach combines two basic formulations, taking advantages of both of them—from the Eulerian: adapted stationary mesh that allows improving the accuracy of metal flow prediction significantly and reduces simulation time, and from the Lagrangian: dynamically movable mesh to animate the profile flow after the bearing exit. These features, consolidated by coupled thermal and mechanical tasks available in the software, allow the obtaining of a precise distribution of the profile velocities that, in turn, means the accurate prediction of parameters defining dimensional accuracy of the extrudates.

The first step in the numerical modelling FEM was preparation of proper 3D models of tools and billet using CAD Solid Works program. The geometry of the porthole die has a crucial importance from the point of view of minimum deformation resistance in the extrusion process, which, in turn, relates to minimisation of extrusion force, elastic deflection of the die, improvement in metal flow, dimensional accuracy of the profile and maximisation of the exit speed.

Three different solutions of 2-hole die geometry were taken to calculation—the die with maximum opening of the inlet channels (die 1), conventional porthole die for 6xxx alloys based on the local 3-armed bridges (die 2) and die 3, which is a modified version of die 2. The geometry of die 2 was taken from extrusion industry practice for 6082 alloy. The final design of extrusion die 3 was developed based on gradual optimization of different die geometry using FEM numerical simulations based on three assumed criteria. The first criterion defined a maximum force not exceeding 25 MN. The second optimization factor minimized the elastic deflection of the die during the process, not exceeding 0.5 mm. The third criterion included ensuring an even outflow of metal from the die bearing, so the velocity deviation did not exceed ±20% of the average value. In total, almost 100 numerical FEM simulations were carried out analysing the influence of portholes, webs, cores, welding chambers, pockets and bearing geometry to determine the final specific die geometry.

**Figure 2:**
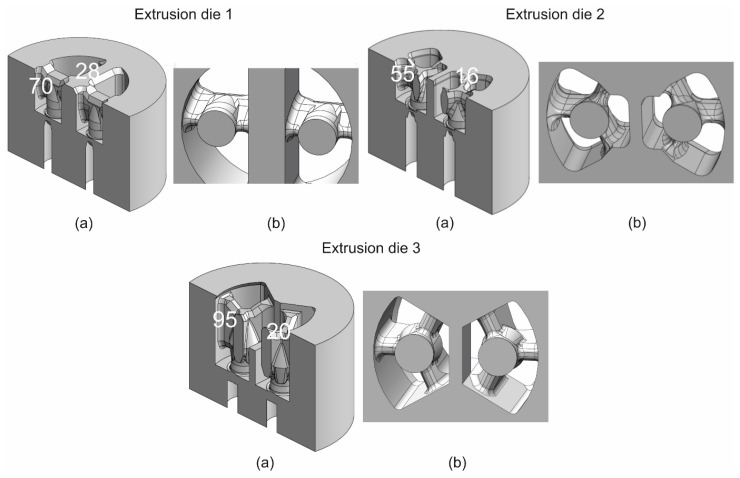
The 3D CAD models of different porthole dies for extrusion of tubes of Ø50 × 2 mm from 7021 alloy: (**a**) in the cross-sectional view and (**b**) in the bottom view—with the bridge dimensions marked (height and thickness).

The massive bridges were assumed for die 1 and adapted for hard 7xxx alloys; the thickness of bridges for die 3 was increased by 4 mm, whereas their length was increased by 40 mm in relation to die 2. In addition, a radial and convex entry to cavity of die 3 was added. The thickness of bridges was equal to 28, 16 and 20 mm for die 1, die 2 and die 3, respectively; length of bridges was 70, 55 and 95 mm, whereas height of the welding chambers was 25, 30 and 27 mm. The maximal broad inlet channels, shaped pockets, bearings of varied length and proper geometry of the central baffle and mandrels were adapted for die 3. The 3D models of the porthole dies discussed and the view from the bottom are presented in Figure 2a,b.

**Figure 3:**
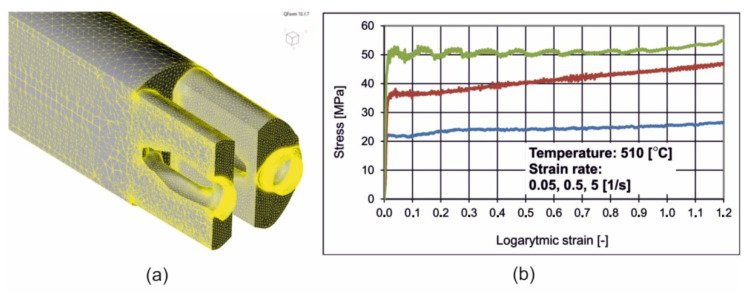
The geometrical model of extruded material with mesh elements (**a**) and determined material model for FEM calculations—dependence of flow stress vs. logarithmic strain for different strain rates for alloy 7021 (**b**): blue colour for 0.05 1/s, red colour for 0.5 1/s and green colour for 5 1/s.

The second step of the FEM modelling was preparation and implementation of the material model for the alloy 7021, which after approximation was expressed by the constitutive equation of Hansel–Spittel (Equation (1)). The starting point was the plastometric compression tests with the use of Gleeble 3800 simulator (Dynamic Systems Inc., Poestenkill, NY, USA), which allowed for determining stress–true strain equation, dependent on temperature and strain rate (Figure 3).

(1) $$\sigma =1090e^{-0.0675T}\cdot T^{0.056}\cdot \varepsilon^{-0.055}\cdot e^{\frac{-0.015}{\varepsilon }}\cdot {\left(1+\varepsilon \right)}^{0.019T}\cdot e^{-0.026\varepsilon }\cdot {\dot{\varepsilon }}^{0.21}\cdot {\dot{\varepsilon }}^{-0.00019T}$$

where σ—plastic stress, ε—plastic strain, $\dot{\varepsilon }$—strain rate, T—temperature of deformation.

The third step of the FEM modelling was defining of boundary conditions. Friction model used in QForm allowed for taking into account the adhesion effect between aluminium and steel. The constant shear friction law is adapted everywhere between workpiece and tool materials, except in bearings where the contact pressure is relatively low. The friction on the bearings was calculated based on the phenomenological model that includes a number of parameters, such as resulting bearings angle, pressure, etc. This approach allowed us to obtain the realistic behaviour of the metal in simulation with three possible zones: sticking, sliding, and separation zone. All the defined extrusion process parameters are presented in Table 4.

### 2.3. Extrusion Trials of Round Tubes

The extrusion trials for the round tube of Ø50 × 2 mm from the 7021 alloy with the use of the discussed porthole dies were performed on the hydraulic direct press of 25 MN capacity and equipped with container of 7″ in diameter.

**Figure 4:**
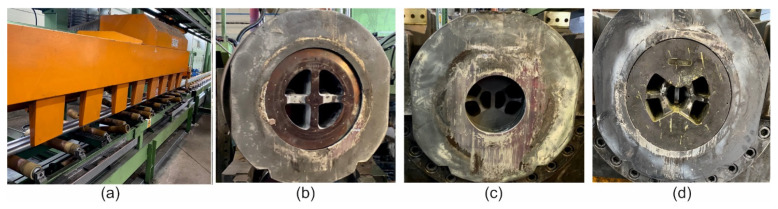
The 25 MN 7 inch extrusion press run-out table (**a**) and different extrusion porthole dies: extrusion die 1 (**b**), extrusion die 2 (**c**) and extrusion die 3 (**d**)—designed for 2-hole extrusion tests of tubes of Ø50 × 2 mm from 7021 alloy in industrial conditions.

The conditions of the trials were identical to those in the numerical simulation for different billet heating temperature and extrusion speed. In the case of extrusion through die 3, the trials were performed for billet temperature of 480 °C and ram speed of 1.5 mm/s as a result of the experiences gained during previous extrusion with dies 1 and 2, which were also tested with higher extrusion speeds and higher billet temperatures. The run-out table of the press with the cooling system and the tested porthole dies of two holes are shown in Figure 4. Extruded tubes were next submitted to stretching with the true strain equal to 0.5%.

During the trials, the following process parameters were recorded: ram speed, exit speed, extrusion pressure and profile temperature with the use of the data acquisition system.

The surface quality was inspected on-line with respect of cracking or tarnish. The maximal extrusion speed was recorded when cracking on profile surface began. The samples taken from the tubes extruded with different process conditions were submitted to optical scanning to check their dimensional accuracy. Similar optical scanning was performed on the used dies and their dimensions were compared with these for new ones.

### 2.4. 3D Optical Scanning of Extruded Tubes and Dies

**Figure 5:**
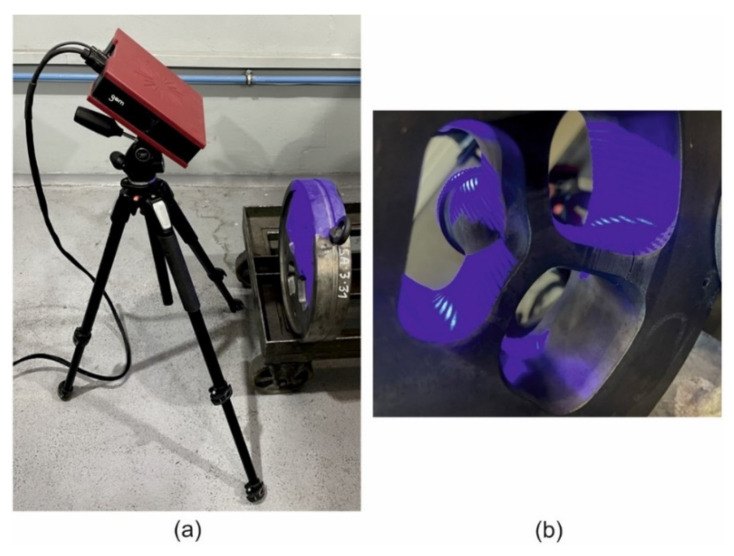
The scanner GOM Atos Core 200 for optical measuring the geometry of extruded tubes and dies (**a**) and the scanned extrusion porthole die (**b**).

The scanner GOM Atos Core 200 (Lenso, Poznań, Poland) (Figure 5a) was used for scanning of the extruded elements on the inner and outer surface of the samples to measure diameters of the tubes, deviations from circularity and wall thickness. The surface was cleaned off from the impurities before scanning. The coloured map of deviations was also obtained on the basis of the CAD model and scanned element.

The first step of the dies measurements included gathering coordinates of all the selected points on the samples’ surface. Next, the detailed scanning from the different perspectives was realized to digitalize the whole available surface. Due to complicated shape of the dies, the process needed about 75 scans from each side. The surfaces of bridges, mandrels and bearing lands were inspected, in particular. The coloured maps of deviations allowed indications of the regions evidently subjected to wearing.

### 2.5. Statistical Analysis of Results for Extruded Tubes and Extrusion Dies

The analysis of the results was performed with the use of Gom Inspect program, and the deviations from the nominal geometry were generated. The deviations concerned diameters, ovality, eccentricity, wall thickness and surface quality of the extruded tubes. The results were analysed and the statistical correlations was obtained using Origin Lab code. The correlation determined interactions between selected variables but did not explain the reasons or the method of their origination. A positive correlation (correlation coefficient from 0 to 1) suggested that as one feature rose, the mean values of the second increased too. A negative correlation (correlation coefficient from −1 to 0) indicated the opposite dependence. The power of correlation is defined in the following way: 0.2 poor correlation; 0.2 ÷ 0.4 low correlation (distinct dependence); 0.4 ÷ 0.6 moderate correlation (key dependence); 0.6 ÷ 0.8 high correlation (significant dependence); 0.8 ÷ 0.9 very high correlation (very high dependence); 0.9 ÷ 1.0 full dependence. In the case of dies, the results were converted to ANOVA statistics.

## 3. Results

### 3.1. FEM Numerical Calculations

**Figure 6:**
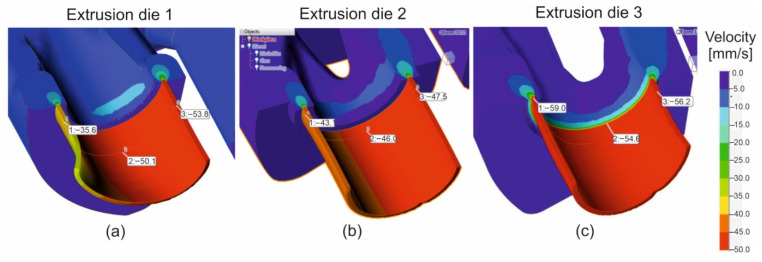
Distribution of metal velocity during extrusion of tubes of Ø50 × 2 mm from 7021 aluminium alloy through porthole dies of different geometry—die 1 (**a**), die 2 (**b**) and die 3 (**c**).

Figure 6 presents the distributions of metal particles velocity during the extrusion of tubes. The metal flows from the inlet channels of the porthole die via welding chambers up to die opening—for design of die 1 (Figure 6a, on the left), die 2 (Figure 6b in the middle) and die 3 (Figure 6c, on the right). Some non-uniformity of metal flow while extruding through die 1 (Figure 6a) can be observed, which manifests itself by the diversification of particles velocity in the die cavity on the tube perimeter. In the outside regions of the die opening, the particles velocity is as high as 35.6 mm/s and is lower by about 31% in relation to the mean value, whereas in the inside regions of the die cavity (and simultaneously close to the central axis of extrusion) it is 53.8 mm/s and is higher by about 16% in relation to the mean value. Some lower values of the particles velocity were observed for die 2, equipped with two local three-dorsal bridges; however, such die design, based on three points feeding of the die opening, provided more uniform distribution of metal particles on the perimeter of the extruded tube in comparison to die 1 (Figure 6b). In the outside region of the die cavity, the particles velocity is as high as 45.1 mm/s (in case of die 1 was 35.6 mm/s) and is only around 5% lower than the mean values, whereas in the inside regions of the die cavity it is 47.5 mm/s and is higher by about 4% in relation to the mean value. The highest and the most uniform metal exit speed was observed in the case of die 3 (Figure 6c). The average metal exit speed for die 3 is about 25% higher compared to that of die 2, as a result of applying the radial and convex entry to the die cavity and original geometry of a central baffle wall regulating the metal flow.

**Figure 7:**
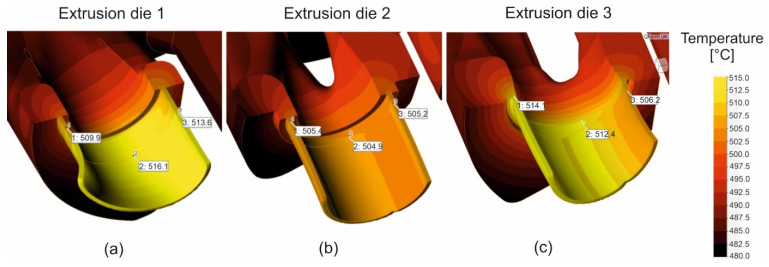
Distribution of metal temperature during extrusion of tubes of Ø50 × 2 mm from 7021 aluminium alloy through porthole dies of different geometry—die 1 (**a**), die 2 (**b**) and die 3 (**c**).

Figure 7 presents the distribution of temperature of the metal during extrusion for the design 1 of die (Figure 7a—on the left), design 2 (Figure 7b—in the middle) and design 3 (Figure 7c—on the right). The higher values of metal temperature of about 9–10 °C were predicted in the case of die 1 and die 3 (516.1 °C and 514.1 °C, in relation to 505.2 °C). The reason for this can be because of the existence of some higher mean velocities of particles and the higher extent of plastic deformation and its conversion into heat.

**Figure 8:**
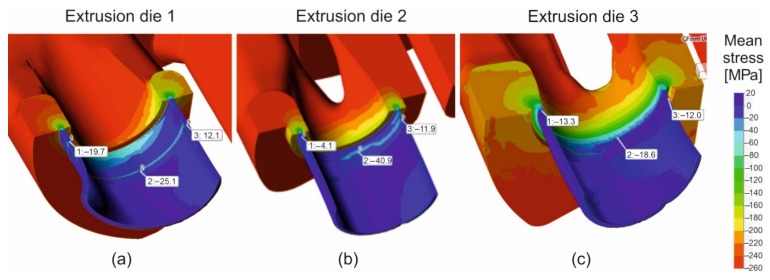
Distribution of mean stress during extrusion of tubes of Ø50 × 2 mm from 7021 aluminium alloy through porthole dies of different geometry—die 1 (**a**), die 2 (**b**) and die 3 (**c**).

Figure 8 presents the distribution of means for the design of die 1 (Figure 8a—on the left), die 2 (Figure 8b—in the middle) and die 3 (Figure 8c—on the right). More beneficial states of stress occur for die 2 and die 3; whereas on the whole perimeter of the die land, the compression pressures are observed, favourable for the deformability of the material and the averages. For die 2, compressive stresses are higher in comparison to die 1 (maximum value of the compression pressure is 40.9 MPa in relation to 25.1 MPa). The most uniform compressive stresses in the die orifice on the extrudate perimeter are observed for die 3. In the case of die 2, within the inside region of the tube (die land of the die opening), positive values of pressure appear at the level of 12.1 MPa, which, in practice, can limit the exit velocity from the die opening.

**Figure 9:**
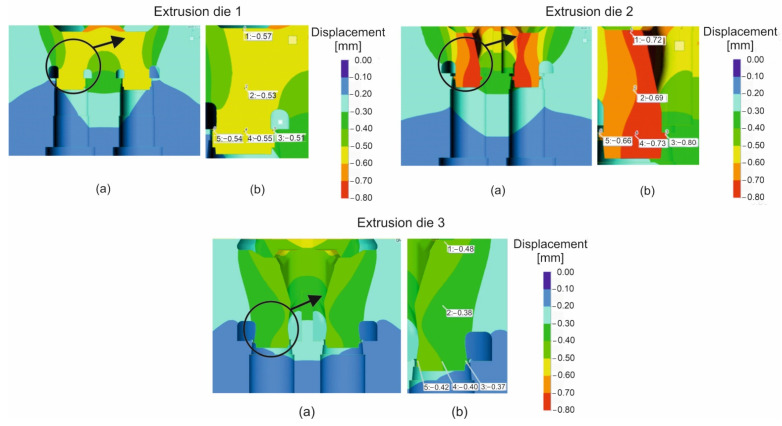
Distribution of die deflection during extrusion of tubes of Ø50 × 2 mm from 7021 aluminium alloy through porthole dies of different geometry—global view of mandrels (**a**) and local view of chosen part of mandrel (**b**).

Figure 9 presents the distribution of elastic deflection of the porthole die in the Z direction, particularly within the regions of the mandrel and the die land for all the die geometrical solutions. Coloured maps indicate that the highest dimensional deviations of the mandrel were made in the case of die 2, where an elastic deflection of 0.66 ÷ 0.80 mm was predicted within the area of the die land. In the corresponding area for die 1, the mandrel deflection was much lower and kept itself within the range 0.51 ÷ 0.55 mm. The lowest mandrel deflection of 0.37 ÷ 0.48 mm was predicted for die 3, depending on the mandrel position.

**Figure 10:**
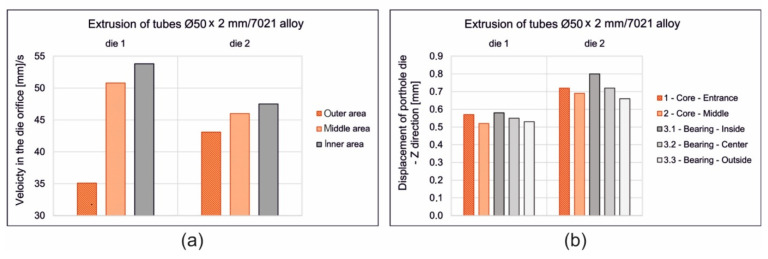
Diagrams of the metal velocity in the die cavity during extrusion of tubes of Ø50 × 2 mm from 7021 aluminium alloy through porthole dies of different geometry (**a**) and porthole die deflection in Z direction in different areas of mandrel for different die geometry (**b**).

The detailed data concerning the metal exit speed in the die cavity on the profile perimeter and elastic deflection in different regions of the mandrel for the all analysed dies are presented in Figure 10. Based on the calculations, it can be concluded that porthole die 3 is the most advantageous and guarantees the highest and most uniform metal exit speed and the lowest elastic deformation of the mandrels in the extrusion direction. This allows the forecasting of the smallest dimensional deviations of extruded tubes for die 3.

**Figure 11:**
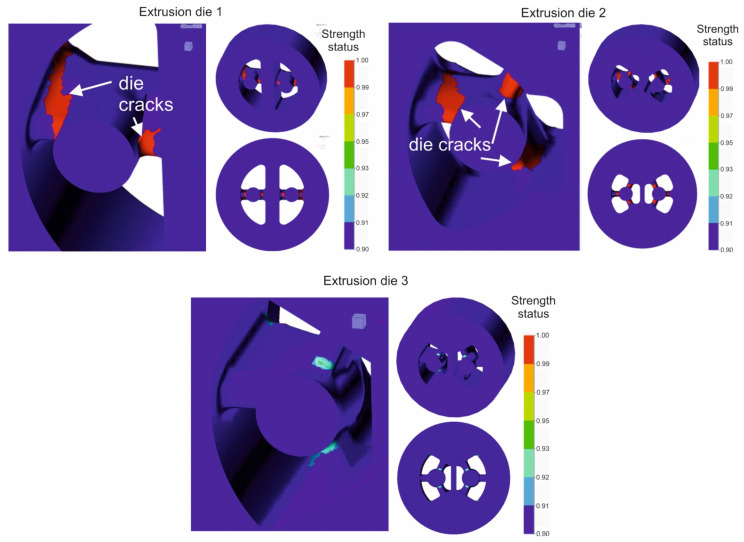
Distribution of strength status for the mandrel tops for different die geometry during extrusion of tubes of Ø50 × 2 mm from 7021 aluminium alloy.

Figure 11 presents the distributions of predicted indicator of the strength status for the porthole dies of different geometry. The indicator of the strength status—red area of the scale—means a high risk of cracking or even mechanical tearing of the die. The maximal values of the indicator for die 1 and die 2 occur in the region of mandrel tops. The significant improvement in the strength conditions in case of die 3 is observed as a result of the elimination of dangerous areas of the bridge arms and the mandrels. For die 3, the indicator of strength status is placed within the range of 0.92–0.95. These benefits result from appropriate changes in the design of the porthole die, i.e., the geometry of the inlet channels, bridges and mandrels.

### 3.2. Extrusion Trials

**Figure 12:**
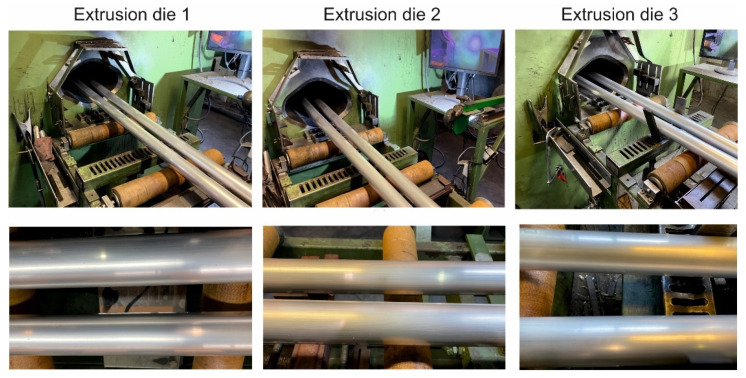
Extruded tubes of Ø50 × 2 mm from 7021 alloy on the press run-out table for dies of different geometry: die 1, T_0_ = 480 °C, V_1_ = 4.5 m/min; die 2, T_0_ = 480 °C, V_1_ = 3.5 m/min; die 3, T_0_ = 480 °C, V_1_ = 4.5 m/min.

**Figure 13:**
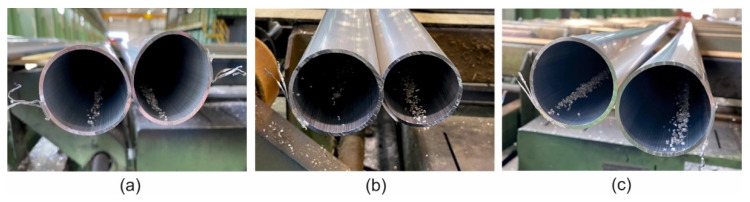
Tubes of Ø50 × 2 mm extruded from 7021 alloy in cross-section by using different dies—die 1 (**a**), die 2 (**b**) and die 3 (**c**).

The extrusion of the examined tubes was performed for die 1, die 2 and die 3 for variable heating temperatures of billet: 520 °C, 510 °C, 500 °C and 480 °C and for variable extrusion speed: 1.5 ÷ 4 mm/s. In the case of die 3, the billet temperature was of 480 °C and the extrusion speed was of 1.5 mm/s. Figure 12 presents the tubes on the run-out of the press extruded by using different porthole dies. In general, for the all tested dies, high geometrical stability of the tubes and high quality of the tubes surface, free of tarnish and cracks, can be noticed. The extrusion pressure of 80–90% of the press capacity and exit speed from dies of 4.5 m/min (for die 1), 3.5 m/min (for die 2) and 4.5 m/min (for die 3) were recorded using a data acquisition system. The tubes of Ø50 × 2 mm, extruded from 7021 alloy in cross-section by using different dies, are presented in Figure 13.

**Figure 14:**
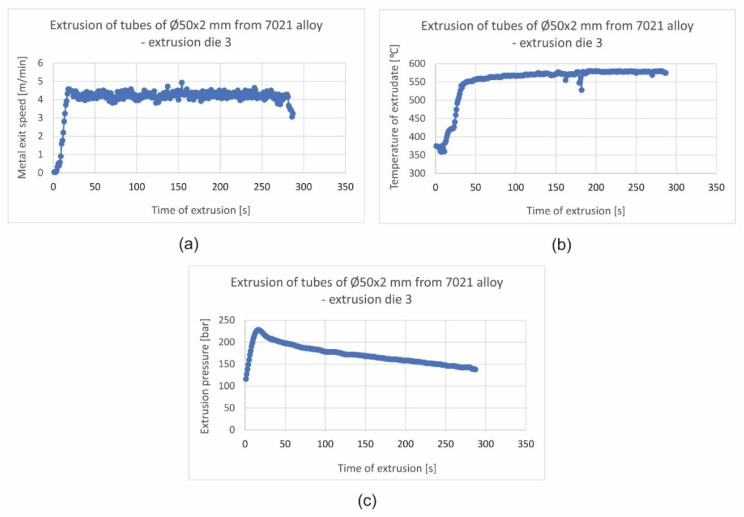
Recorded technological parameters of extrusion of tubes of Ø50 × 2 mm from 7021 alloy for extrusion die 3: metal exit speed (**a**), extrudates temperature (**b**) and extrusion pressure (**c**).

Exemplary technological parameters of extrusion process for die 3 were shown in Figure 14. Experimental trials confirmed the possibility of obtaining a relatively high metal exit speed with good surface quality of profiles for die 3 with radial and convex entry to the die cavity.

**Figure 15:**
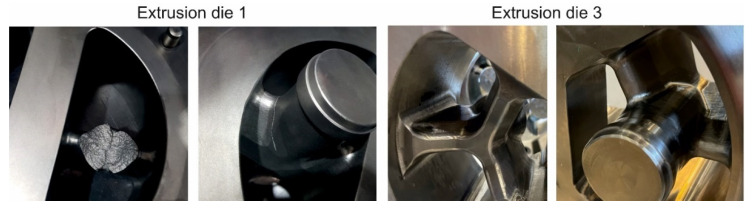
Porthole dies after extrusion of tubes of Ø50 × 2 mm from 7021 alloy; broken mandrel view for extrusion die 1 (on the **left**), bridge and mandrel view for extrusion die 3, free from cracking (on the **right**).

During the tests for die 1 and 2 and for the lowest billet temperature, the cracking of a mandrel occurred in both the tested dies and the mandrels were damaged (Figure 15 on the left). In the case of die 3, bridges and mandrels were free from cracks after extrusion (Figure 15 on the right), which confirmed the earlier FEM predictions. The qualitative assessment of die 3 indicated minimal deflection of the bridges. The dies were submitted to quantitative analysis—3D optical scanning.

### 3.3. 3D Optical Scanning of Extruded Tubes

**Figure 16:**
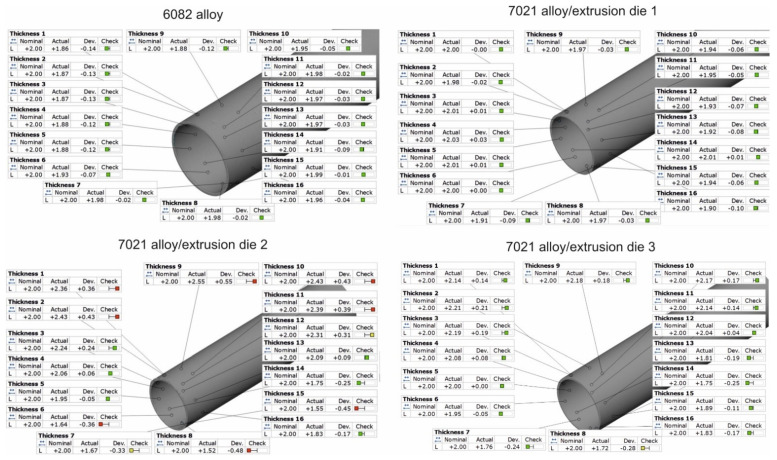
Results of optical scanning of the tubes of Ø50 × 2 mm: measured wall thickness of tubes extruded from 6082 alloy by using 4-hole die (on the **top left**) and extruded from 7021 alloy by using 2-hole die 1 (on the **top right**), 2-hole die 2 (on the **bottom left**), as well as extruded from 7021 alloy by using 2-hole die 3 (at the **bottom right**).

**Figure 17:**
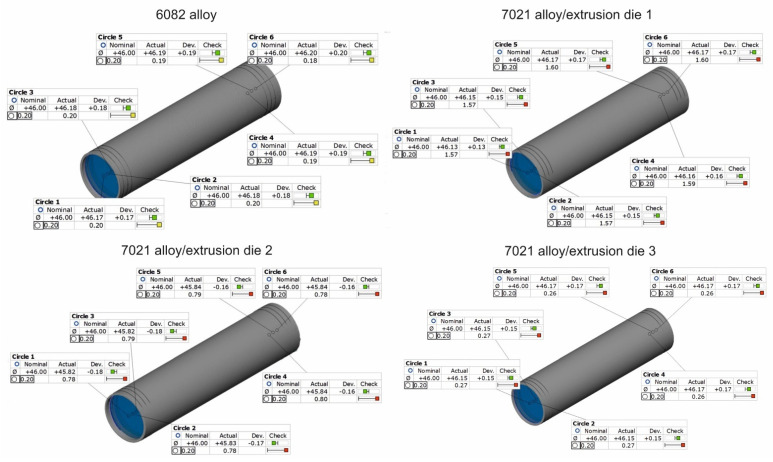
Results of optical scanning of the tubes of Ø50 × 2 mm: measured inner diameter and roundness deviations of tubes extruded from 6082 alloy by using 4-hole die (on the **top left**) and extruded from 7021 alloy by using 2-hole die 1 (on the **top right**), 2-hole die 2 (on the **bottom left**), as well as extruded from 7021 alloy by using 2-hole die 3 (on the **bottom right**).

Figure 16 and Figure 17 present the results of the optical scanning with the use of a ATOS Core 200 system, purchased from the GOM Company, for tubes of Ø50 × 2 mm extruded from 7021 alloy through the porthole dies: 1, 2 and 3 and, for comparison, through the tubes of identical geometry from 6082 alloy extruded through the classic porthole die for 6xxx series alloys. Figure 16 refers to the measurements of wall thickness in 16 points along the perimeter of the tube, whereas Figure 16 shows the measurement results of the inner diameter of the tube and deviations from the circularity in six points along the perimeter of the tube. The figures also present results of the inspection of the dimensional accuracy of the wall thickness, inner diameter and circularity of the extruded tubes in comparison with permissible deviations from the nominal dimensions described in the standard. The green colour means that a given dimension fits permissible limits in the standard, the yellow colour means that a dimension is placed on the edge of the standard and the red colour means that the dimension is out of the standard.

On the basis of the measurements, a high mapping result was obtained for the wall thickness, inner diameter and circularity for tubes extruded from the 6082 alloy at all the measurement points in which the dimensional deviations were acceptable (Figure 16 on the left and Figure 17 on the left). In the case of tubes from the 7021 alloy extruded through die 1 and 2, the relatively high dimensional deviations of wall thickness and circularity were observed, with the simultaneous preservation of narrow deviations of outer and inner diameters. The maximal deviations of the wall thickness reached 0.48 mm (Figure 16, die 2), which, together with concurrent positive deviation at the level of +0.43 mm, leads to a strong tube eccentricity phenomenon. However, maximal circularity deviations reached as much as 1.6 mm (Figure 16, die 1), which considerably exceeds an acceptable deviation of circularity, defined at the level of 0.25 mm. At the same time, for die 1 the narrow dimensional tolerances of the wall thickness for the tube from the 7021 alloy occurred and were better in relation to the tube from the 6082 alloy, maximal measured deviations did not exceeded 0.1 mm and permissible deviation was at the level of ±0.25 mm. At the same time, the use of die 3 is associated with a radical improvement in the dimensional tolerances of the extruded tubes—the average deviation of wall thickness was 0.14 mm, the average deviation of circularity was already acceptable at the level of 0.27 mm and the average deviation of centricity was 0.25 mm.

**Figure 18:**
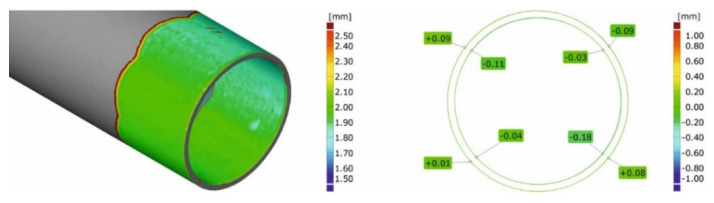
Results of optical scanning of the tubes of Ø50 × 2 mm from 6082 alloy: coloured maps of deviations for 6082 alloy using a 4-hole die (wall thickness on the **left** and roundness/circularity deviations on the **right**).

**Figure 19:**
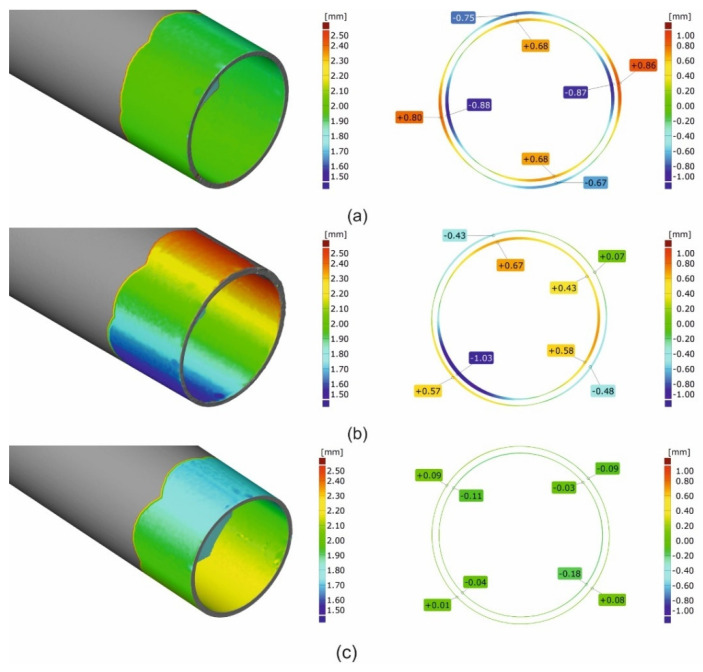
Results of optical scanning of the tubes of Ø50 × 2 mm: coloured maps of deviations for tubes extruded from 7021 alloy with using 2-hole die 1 (**a**), 2-hole die 2 (**b**) and 2-hole die 3 (**c**) (wall thickness on the left and roundness deviations on the right).

**Figure 20:**
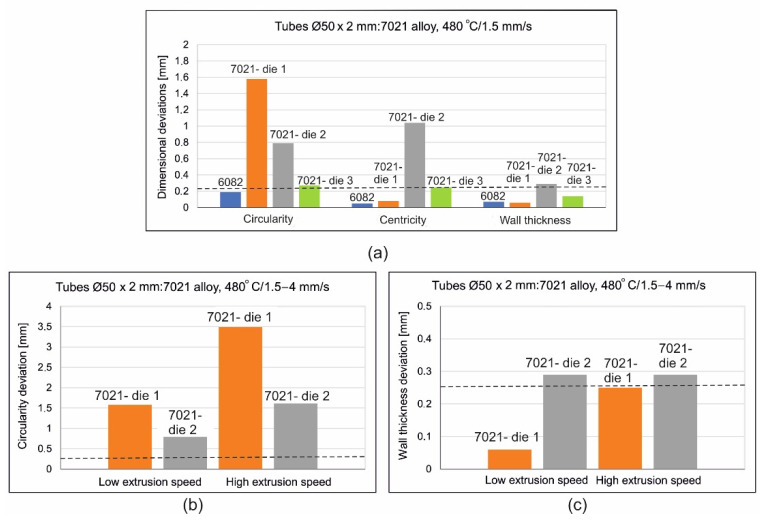
Results of optical scanning of the tubes of Ø50 × 2 mm extruded from 6082 alloy through 4-hole die and from 7021 alloy extruded through die 1, die 2 and die 3, (**a**)—comparison of deviations for tubes extruded from 6082 and 7021 alloys, (**b**)—circularity deviations for 7021 alloy, (**c**)—wall thickness deviations for tubes from 7021.

The results described above are illustrated in Figure 18 and Figure 19 in the form of coloured maps of deviations, as well as in Figure 20, where the deviation of circularity, eccentricity and wall thickness for both the analysed dies are jointly shown in comparison to the standard (dashed line). In addition, an influence of the extrusion speed (within the range of 1.5 ÷ 4 mm/s) on dimensional deviations of tube for dies 1 and 2 in Figure 20 is presented. In particular, a strong influence of the extrusion speed on the circularity deviation for die 1 can be observed. This is connected with the occurring of the considerable ovality of the tube at relatively high extrusion speeds for the 7021 alloy.

The influence of extrusion speed on the level of circularity deviation for die 2, which rises from 0.8 mm up to nearly 1.5 mm (as extrusion speed changes within the range of 1.5 ÷ 4 mm/s), is also significant. Regarding the eccentricity of the tube in the case of die 1, a low acceptable value is observed even for high extrusion speeds. However, a large value of eccentricity for the tube of die 2 is observed (over 1 mm) for a low extrusion speed of 1 mm/s, which unexpectedly drops twice for high extrusion, with a speed of 4 mm/s. As reported before, a small deviation of the wall thickness below 0.1 mm for die 1 and a low extrusion speed of 1.5 mm/s occurred, whereas for a high extrusion speed of 4 mm/s the deviation slightly exceeds the permissible value of ±0.25 mm. In the case of die 2, a large deviation of the wall thickness, exceeding the acceptable limit is observed, independently of the extrusion speed (Figure 20). Die 3 provided the lowest and most acceptable geometrical deviations of extruded tubes from the 7021 alloy.

### 3.4. Corelation Statistical Analysis of Extruded Tubes

**Figure 21:**
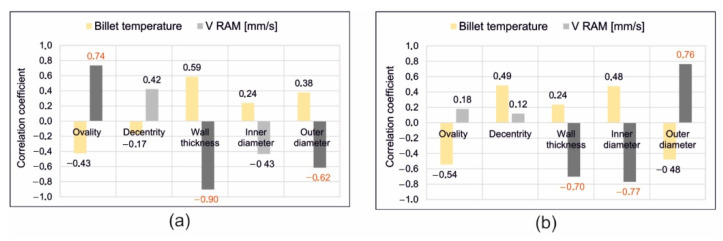
Results of statistical correlation analysis for optically scanned tubes of Ø50 × 2 mm extruded from 7021 alloy through porthole dies for different billet temperatures and different ram speeds, (**a**) die 1, (**b**) die 2.

Based on the analysis results, a correlation coefficient was established. In particular, a coefficient from 0.6 to 1.0 (high, very high and full correlation) was taken into consideration. The significant coefficients of correlation were indicated in red in Figure 21. A strong correlation of variables, such as extrusion speed, circularity, wall thickness, inner diameter, and outer diameter of a tube, and a moderate correlation of variables, such as billet temperature and centricity, was found. In particular, for die 1 a high correlation of extrusion speed and centricity of the tube (coefficient of 0.74) was obtained, which means that the rise in extrusion speed within the range of 1–4 mm/s leads to high increase in the tube centricity. In turn, a very high negative correlation between extrusion speed and wall thickness (−0.90), as well as between outer diameter (−0.62), indicates that the increase in the extrusion speed leads to significant decreasing of both wall thickness and outer diameter of the tube. The rise in temperature moderately influences the decrease in centricity of the tube (correlation coefficient of −0.43).

In the case of die 2, a negative and high correlation of the extrusion speed and wall thickness is observed (correlation coefficient of—0.70), as well as the extrusion speed—inner diameter (correlation coefficient of—0.77), whereas a high correlation between extrusion speed and the outer diameter (coefficient of correlation 0.76) occurred. This means, in practice, a high decrease in the wall thickness and inner diameter, as well as an increase in the outer diameter, will cause a rise in extrusion speed. Moderate decreases in the circularity of the tube with an increase of billet temperature (correlation coefficient of—0.54) is also visible.

**Figure 22:**
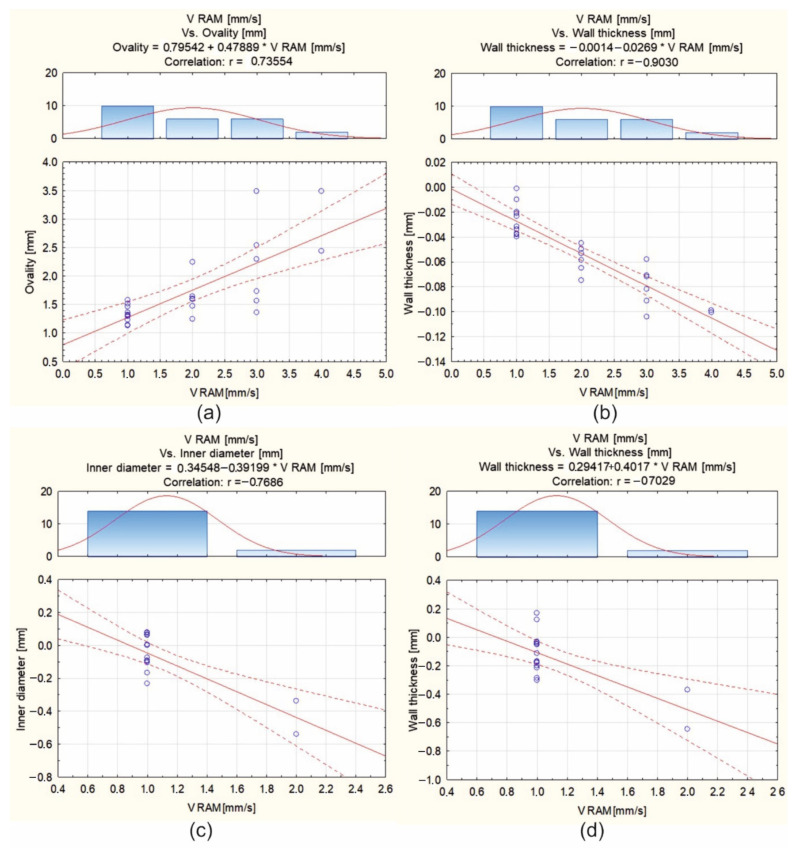
Scatterplots for optically scanned tubes of Ø50 × 2 mm extruded from 7021 alloy through porthole dies for different billet temperatures (480 °C, 500 °C, 510 °C and 520 °C) and different ram speeds (1.5, 2, 3 and 4 mm/s): die 1—ovality and wall thickness deviations (**a**,**b**), and die 2—inner diameter and wall thickness deviations (**c**,**d**).

Figure 22 presents the scatter diagrams of results for the tube extrusion of Ø50 × 2 mm from 7021 alloy through die 1 (Figure 22a,b; correlation of extrusion speed and ovality, as well as wall thickness) and through die 2 (Figure 22c,d; correlation of extrusion speed and inner diameter, as well as thickness of the tube).

### 3.5. 3D Optical Scanning of Extrusion Porthole Dies

**Figure 23:**
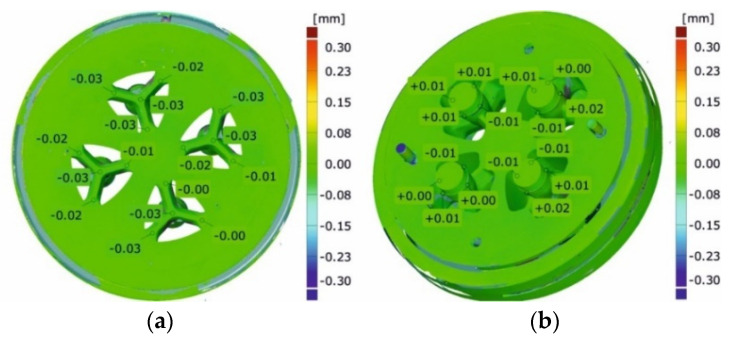
Results of optical scanning of the porthole dies for extrusion of tubes of Ø50 × 2 mm from 6082 alloy: die deflection—bridges view (**a**) and the mandrels (**b**).

**Figure 24:**
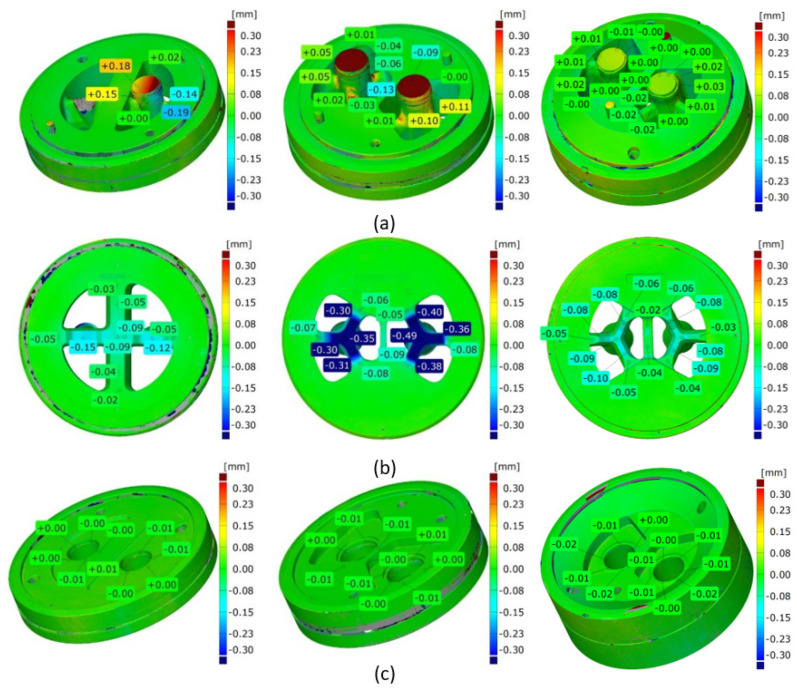
Results of optical scanning of the porthole dies for extrusion of tubes of Ø50 × 2 mm from 7021 alloy: die deflection for mandrels (**a**), bridges (**b**) and inserts (**c**)—die 1 (on the left), die 2 (in the middle) and die 3 (on the right).

Figure 23 shows the maps of dimensional deviations of the porthole die after extrusion of tubes of Ø50 × 2 mm from the 6082 alloy (bridges view—on the left, mandrel view—on the right). It should be noted that there are minimal dimensional deviations of bridges, both in the central part and on the bridge arms, not exceeding 0.03 mm, indicating a small elastic deflection in the extrusion direction and finally, on a small permanent deformation during the extrusion process. A similar situation exists for mandrels, particularly in the region of bearing length, where dimensional deviations are small at the level between -0.01 mm and 0.03 mm. Analogical maps for porthole dies after the extrusion of tubes from the 7021 alloy are presented in Figure 24.

Figure 24a shows mandrels, Figure 24b—bridges and Figure 24c—die inserts (die 1 on the left, die 2 in the middle, and die 3 on the right). The significant dimensional deviations of mandrels for die 1 and die 2 are visible (Figure 24a on the left and in the middle). Slightly higher deviation for die 1 is observed from −0.19 mm at the outer part of the mandrel up to +0.18 mm at the inner part of the mandrel. This suggests permanent deviation of the mandrel to the centre of the die; the tearing of one mandrel region is also visible. The situation for die 2 looks in reverse, i.e., there is a permanent deviation of mandrels outside the die, whereby the dimensional deviations are smaller compared to die 1. Very small dimensional deviations of the mandrels, not exceeding 0.3 mm, were stated for die 3 (Figure 24a on the right). A moderate deflection of bridges in the extrusion direction for die 1 is observed (Figure 24b on the left)—deviation is at the level of −0.12 mm down to −0.15 mm, whereas the significant deflection of the bridges for die 2 was stated at the level between −0.35 and −0,49 (Figure 24b in the middle). The minor bending of the bridges in the extrusion direction, not exceeding −0.1 mm, was stated for die 3 (Figure 24b on the right). In practice, zero dimensional deviations were stated for regions of die lands of all the porthole dies (Figure 24c).

**Figure 25:**
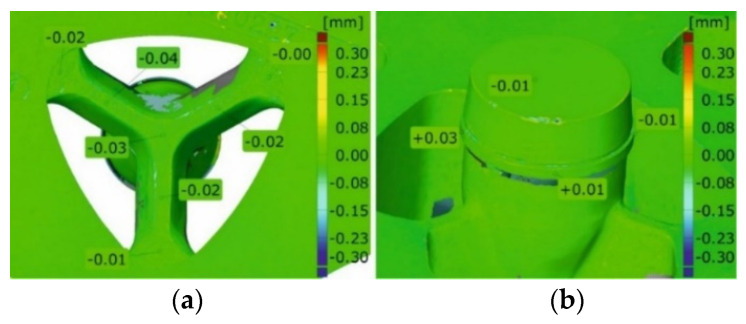
Results of optical scanning of the porthole dies for extrusion of tubes of Ø50 × 2 mm from 6082 alloy: die deflection—close-up of the bridge view (**a**) and the mandrel view (**b**).

**Figure 26:**
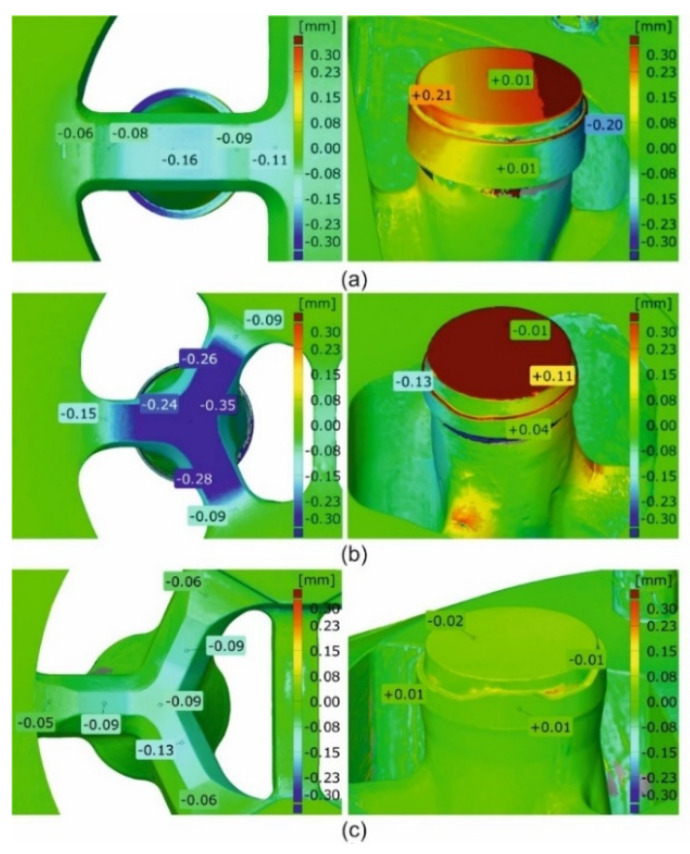
Results of optical scanning of the porthole dies for extrusion of tubes of Ø50 × 2 mm from 7021 alloy: die deflection—close-up of view for die 1 (**a**), die 2 (**b**) and die 3 (**c**).

The close-up of a chosen bridge (on the left) and a chosen mandrel (on the right) for the discussed tube from the 6082 alloy in Figure 25 is presented. Figure 26 presents the close-up of the chosen bridges (on the left) and the chosen mandrels (on the right) for the extrusion of discussed tubes from 7021 alloy—for die 1 (Figure 26a), die 2 (Figure 26b) and die 3 (Figure 26c).

### 3.6. ANOVA Statistical Analysis of Extrusion Porthole Dies

The results from the measurements with the use of optical scanning 3D were utilized in a statistical analysis. Surfaces of the bridge part and insert die were divided between the regions in which the dimensional deviations were determined, which was the result of the matching CAD models of the dies and real scans.

**Figure 27:**
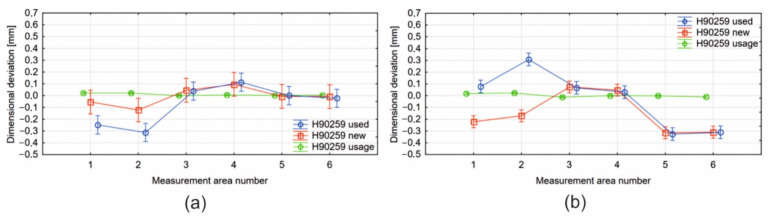
Results of ANOVA analysis for the porthole die 1: die core/bridge part (**a**) and die disc/insert (**b**).

In the case of the bridge-part of die 1 (H90259), three crucial areas were analysed. Area 1 relates to the frontal part of the bridge. It was considered as a whole without demarking between particular apertures because of the die design, areas 2–5 concern the side surface of the channel and areas 6–7 indicate the surface of the mandrel/core land (Figure 27a). The lack of area 7, which marks the surface of the calibrating land on the core, is caused by its damage. In the case of this element, the largest differences, at the level of −0.3 mm, are visible for areas 1 and 2. This is due to the significant deformation of this part of the porthole die during use. The deformation of area 1 results from the bending of the die bridges and area 2 from the excessive wear of the side surface of the channel.

In the case of the insert-part of die 1 (H90259), three crucial areas were analysed. Areas 1–2 indicate the frontal part of the channel, 3–4 the side surface of the channel and areas 5–6 relate to the die land. In the case of this element, the highest deviation values, at a maximum level of −0.3 mm, occur for the areas 5–6 (Figure 27b). The obtained values result from insufficient precision in the workmanship of the element, since the diagram representing wear indicates small deviations from the nominal value.

**Figure 28:**
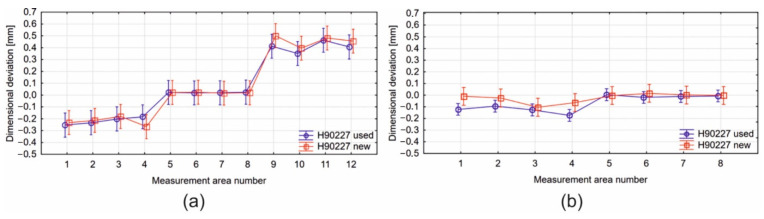
Results of ANOVA analysis for the porthole die 2: die core/bridge part (**a**) and die disc/insert (**b**).

Three regions for each die aperture were marked in the case of the bridge-part of die 2 (H90227). The area marked with 1–4 means the dimensional deviations of the channel surface, 5–8 refers to deviations from the surface of the core land and 9–12 are related to the frontal surface of the bridge (Figure 28a). The highest deviations, at the level of −0.45 mm for the new die and −0.40 mm for the used die, occur for the area 9–12, which means that these regions seem most exposed to wear.

In the case of the insert-part of die 2 (H90227), two areas were marked and analysed. Areas 1–4 indicate deviations obtained from the side surface of channels in the die insert, whereas areas 5–8 relate to quantities obtained from the die land surface (Figure 28b). The results indicate small dimensional deviations at a level of 0.07 mm, whereas the largest differences occur in the case of the side surface of the channel in comparison to the die land values.

## 4. Discussion

The distribution of the metal velocity during the extrusion of the tubes of Ø50 × 2 mm from a hard 7021 alloy, with the use of die 1 (Figure 6a), indicates a significant difficulty in metal supply to the outer regions of the die cavity in the sub-bridge area. In this area, the metal flows over the bridge, then it flows across the welding chamber, before it reaches the die cavity. The fastest flowing was obtained for the inner regions of the die cavity, as well as for the sub-bridge region, but is located close to the central extrusion axis, where, by nature, the metal flows faster. The high diversity in the metal velocity within the die cavity on the perimeter of the tube may cause the formation of the tensile stresses at the die land (Figure 8a). This, in turn, can lead to the surface tearing of the product and, in consequence, to a decrease in the exit speed from the die, which reduces the effectiveness of the process. Moreover, it may result in a decrease in the dimensional accuracy of the tube and, in particular, in the high deviations from the circularity.

It is worth noting that the improvement of the die 1 design, aimed at the acceleration of the metal flow within outer regions of the die aperture in the sub-bridge area and connected with the extending of the welding chambers and lowering of the die lands, has proved to be unsuccessful. On the one hand, the maximum opening of the inlet channels in die 1, along with an application of the central two-ridge bridge, offered a chance for a decrease in the metal flow resistance during extrusion and to an increase in the average exit speed. On the other hand, it resulted in a non-uniform metal flow at the perimeter of the tube and, finally, impeded a loss of geometrical stability of the extruded tubes (Figure 16, Figure 17, Figure 18, Figure 19 and Figure 20).

As shown in the subsequent photogrammetric investigations, a correlation can be found between the tendency to lose roundness when using die 1 and high uniformity of the metal flow within the die aperture. In the case of die 2, where a uniform metal flow was observed (Figure 6b), deviations from the tube ovality were significantly lower (Figure 16, Figure 17, Figure 18, Figure 19 and Figure 20); however, they also exceeded the values permissible by proper standard. The permanent deviation of mandrels from the extrusion axis in the transverse direction is a main reason of the ovality of tubes (Figure 24 and Figure 26). However, there is also a correlation between deviations of the wall thickness—combined with the eccentricity of the tube—an elastic deflection of mandrels in the extrusion direction (Z direction), and the further permanent deviation of mandrels, as well as bridge bending in extrusion direction (Figure 24 and Figure 26). The differences in elastic deflection of the mandrels in the porthole die results from a diverse mandrel design, particularly its thickness and length.

Lower values of the elastic deflection of the mandrel were obtained for die 1, with bridges of 28 mm in thickness and 70 mm in length, whereas significantly higher elastic deflections occurred for die 2 of 16 mm bridge in thickness and of 55 mm in length. In addition, higher permanent deflections of bridges were measured for die 2. Consistently, the higher dimensional deviations of the wall thickness and eccentricity phenomenon of die 2 were confirmed. However, a large ovality of extruded tubes was stated for the extrusion of die 1 as a result of non-uniform metal outflow from the die opening and back bending of mandrels.

The above observations suggest the inadequate porthole die design of die 1 and die 2, which were modified in terms of better metal flow control and higher strength and stiffness. The following corrections were performed to design the extrusion of die 3: slightly lowered/rounded entry to the inlet channels, the original geometry of central die compartment, thicker bridges of up to 20 mm, elongated bridges of up to 95 mm, greater height of the welding chambers of 21–25 mm, elongated mandrels of modified geometry, and radial-convex entry to the die cavity.

**Figure 29:**
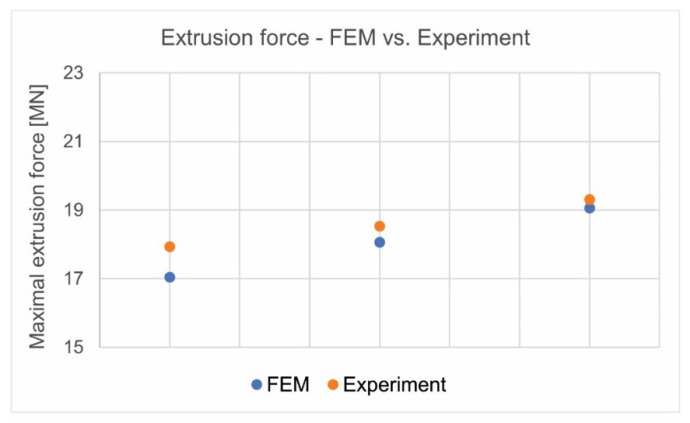
Comparison of extrusion force for the analysed tube from 7021 alloy—FEM predicted and experimentally validated.

The FEM calculations indicated lower elastic deflections of both bridges and mandrels, as well as more uniform supply of the metal to the die aperture, while also eliminating regions where the material passes from the elastic to plastic state (the potential places of the mandrel cracking). The industrial trials of the extrusion of the discussed tubes using modified die 3 and the subsequent 3D optical scanning of extruded tubes and used dies positively validated the established assumptions. A high compliance of extrusion force was obtained for FEM numerical calculations and experimental extrusion tests of the analysed tubes (Figure 29).

## 5. Conclusions

Based on the obtained results, the following conclusions can be formulated:

1. The extrusion of tubes of Ø50 × 2 mm from the 7021 alloy with the use of the porthole dies is connected with high deformation resistance and high frictional resistance in comparison to the extrusion of analogical tubes from the 6082 alloy, which translates to higher extrusion pressures and lower metal exit speeds.
2. High unit pressures during extrusion tubes of Ø50 × 2 leads to high thermo-mechanical loads of dies connected with permanent deflection of bridges and mandrels, which leads to significant dimensional deviations of circularity and eccentricity, exceeding standard’s limits.
3. Strong plus correlation between extrusion speed and tube circularity, strong minus correlation of extrusion speed and the wall thickness, inner and outer diameter of tubes, and moderate minus correlations between billet heating temperature and circularity of tubes exist. This means that maximal permissible extrusion speed cannot be applied, but high billet heating temperatures should possibly be advised.
4. The application of the modified die 3 resulted in successful control of the metal flow, which consisted in the effective facilitating of the inflow of metal to the sub-bridge areas, thus minimizing the side deviation of mandrels.
5. The design of the porthole dies for the extrusion of the 7xxxx series aluminium alloys needs new assumptions in relation to classic dies for the extrusion of the 6xxxx series alloys. In the extrusion of tubes from a 7021 alloy, we recommend: slightly thicker and considerably elongated bridges, special central die compartment, higher welding chambers, shaped pocket dies, geometrically modified mandrels and smooth entries to the die cavity.

## Figures and Tables

**Table 1:** Chemical composition of 6082 alloy in mass percentage.

Alloy Denotation	Si	Fe	Cu	Mg	Cr	Zn	Ti	Zr
6082	1.3	0.21	0.03	0.59	0.00	0.4	0.2	0.1

**Table 2:** Chemical composition of 7021 alloy in mass percentage.

Alloy Denotation	Si	Fe	Cu	Mg	Cr	Zn	Ti	Zr
7021	0.08	0.21	0.00	2.12	0.00	5.47	0.01	0.15

**Table 3:** DSC test results of homogenized 7021 alloy [22].

Alloy	Solidus Temperature, °C	Incipient Melting Heat, J/g
7021 alloy	572.1	0.29

**Table 4:** All the defined parameters of the FEM modelled extrusion process of tubes of Ø50 × 2 mm from 7021 alloy.

Alloy	7021
Billet dimensions	Ø178 × 800 mm
Billet temperature	480, 500, 510, 520 °C
Container/Die temperature	450 °C
Extrusion ratio	42
Stem velocity	1.5–4 mm/s
Metal exit speed	2.5–10 m/min
Friction coefficient	1

## Data Availability

Not applicable.
